# The impact of racism on subsequent healthcare use and experiences for adult New Zealanders: a prospective cohort study

**DOI:** 10.1186/s12889-023-17603-6

**Published:** 2024-01-09

**Authors:** Ricci Harris, Donna Cormack, Andrew Waa, Richard Edwards, James Stanley

**Affiliations:** 1 Eru Pōmare Māori Health Research Centre, University of Otago, Wellington 23a Mein Street, Newtown, Wellington, New Zealand; 2 Department of Public Health, University of Otago, Wellington 23a Mein Street, Newtown, Wellington, New Zealand; 3 Dean’s Department, University of Otago, Wellington, 23a Mein St, Newtown, Wellington, New Zealand

**Keywords:** Racism, New Zealand, Healthcare, Prospective, Cohort study, Unmet need, Healthcare experience, Satisfaction

## Abstract

**Background:**

Racism is an important determinant of health and driver of racial/ethnic health inequities. Experience of racism has been linked to negative healthcare use and experiences although most studies have been cross-sectional. This study examines the relationship between reported experience of racism and subsequent use and experience of health services.

**Methods:**

This is a prospective cohort study design. The 2016/2017 adult New Zealand Health Survey (NZHS) provided the sampling frame and baseline data on exposures, health status and confounders. This stand-alone study invited all exposed individuals to participate when sampled based on their reported experience of racism (ever), stratified by broad ethnic groupings (Māori, Pacific, Asian, European/Other). Equal numbers of unexposed participants were selected for invitation using propensity score matching (propensity to experience racism, based on key available predictive factors). Follow-up was one to two years after NZHS interview. Outcome variables (last 12 months) were: unmet healthcare need (overall, for mental health, for a general practitioner); satisfaction with usual medical centre; and experiences with general practitioners (explaining care, involvement in decision-making, treated with respect/dignity, confidence and trust). Logistic regression models examining the association between experience of racism (at baseline) and health service use and experience (at follow-up) used doubly-robust estimation to weight for propensity scores used in the sampling with additional adjustment for confounders.

**Results:**

The study had 2010 participants. Experience of racism (ever) at baseline was associated with higher overall unmet need at follow-up (adjusted OR (aOR) = 1.71, 95% CI 1.31, 2.23), with similar patterns for other unmet need measures. Experience of racism was associated with higher dissatisfaction with a usual medical centre (aOR = 1.41, 95% CI 1.10, 1.81) and with higher reporting of negative patient experiences.

**Conclusion:**

In line with how racism structures oppression, exposure to racism is largely felt by non-European groups in Aotearoa New Zealand. Experiences of racism potentially lead to poorer healthcare and healthcare inequities through higher unmet need, lower satisfaction and more negative experiences of healthcare. The health system has a critical role to play in addressing racism within healthcare and supporting societal efforts to eliminate racism and ethnic inequities.

**Supplementary Information:**

The online version contains supplementary material available at 10.1186/s12889-023-17603-6.

## Background

Healthcare is an important driver of health outcomes. Differential access to and quality of healthcare are also important determinants of inequitable health outcomes [1]. Stark ethnic health inequities for Māori (Indigenous peoples) and Pasifika peoples in Aotearoa New Zealand (NZ) continue to persist across most health indicators (including healthcare and health outcomes) compared to other New Zealanders, especially compared to the NZ European ethnic group (e.g. [2–4]).

Racism is well recognised as an important ‘basic’ determinant of health that creates and maintains ethnic health inequities, both in NZ and internationally [5–7]. Racism is an organised system that incorporates the idea of ‘race’, and involves ideologies of inferiority and superiority by race/ethnicity that are used to (re)produce and justify violence and oppression [8]. Racism systematically structures differences in opportunities, resources and power, creating disadvantage for some racialised groups, and privilege for others [1, 7]. Entrenched systems of racism have important historical contexts with ongoing manifestations that perpetuate inequity [5, 6]. In NZ and globally, there is an intimate relationship between racism and colonialism that continues to be reflected in health outcomes and healthcare inequities by ethnicity [5, 6]. Racism operates to impact health through multiple pathways and at multiple levels. This includes through the structuring of the healthcare system and differential access to and quality of healthcare [1, 6, 9].

Racism impacts healthcare both directly and indirectly [1, 9]. For example, ethnic inequities in socioeconomic status (SES), reflecting racism within social, economic and political systems, can result in differential access to and quality of care within the healthcare system [1] i.e. better access to and quality of care for NZ Europeans. For example, in NZ, primary care is largely subsidised but still has a patient co-payment for consultation. Māori and Pasifika peoples, who experience more socio-economic disadvantage, are more likely to report unmet need for healthcare, with cost given as the main reason [10]. Funding such as *Very Low Cost Access funding*, aimed at reducing cost barriers to primary care for populations with high health needs, has been shown to be insufficient to close this gap [11], and additional cost-related and other barriers to primary care that disproportionately impact Māori and Pasifika peoples remain, including cost of transport, existing debt, ability to get an appointment and time off work [11, 12].

Racism can also act directly to affect the experience of and access to healthcare through racism at the level of individual healthcare providers and organisations [1, 13]. For example, Māori, Pasifika and Asian populations report higher experiences of multiple types of racism in NZ, including experience of racism by a health professional [14]. Experiences of racism, and especially experience of racism by a health professional, have been associated with lower receipt of timely screening for Māori women, higher unmet need, and poorer patient experiences of care, in cross-sectional analyses in NZ [14]. In addition, ethnic bias against Māori has been demonstrated among medical students and linked to differential quality of clinical decision-making in hypothetical clinical scenarios [15]. Racialised stereotypes held by health professionals and victim-blaming discourses regarding Māori and Māori health have also been demonstrated in other research [16–19]. While quantitative research internationally and in NZ has demonstrated links between experience of racism and a range of negative healthcare measures, previous studies have largely been cross-sectional, with reviews of the topic noting the limited longitudinal evidence available [9, 14].

The **aim** of this paper is to examine the relationship between reported experience of racism and subsequent use and experience of health services. We hypothesised that patients who reported experiencing racism would be more likely to experience future unmet healthcare needs and be more likely to report negative healthcare experiences. The study uses a prospective design to better examine the direction of association from racism to healthcare use and experiences.

## Methods

### Theoretical and conceptual approach

The project was informed by critical and transformative research principles, which contextualise the study within a broader recognition of racism as a root cause of inequities linked to colonialism [6, 20]. For example, this includes an understanding that racism and colonialism structure healthcare and broader social environments, and also shape the way ethnicity, and other constructs such as gender, are conceptualised and categorised in health research. The study questionnaire content was informed by a literature review and conceptual models of the links between racism and healthcare [21].

### Study design and sampling frame

This study uses a prospective cohort study design. It was approved by the University of Otago’s Human Ethics (Health) Committee (reference: H17/094). The 2016/2017 New Zealand Health Survey (NZHS) adult respondent dataset was the sampling frame for selecting participants for this cohort study, and also provided baseline data on exposures, health status and confounders. The study followed up a subgroup of NZHS participants from their baseline NZHS interview (Time 1 = T1) to follow-up between 12 and 24 months later (Time 2 = T2). The adult NZHS uses a complex sample design to produce representative results for a cross-section of New Zealand adults aged 15 + years and is managed by the New Zealand Ministry of Health [22].

Full details on the study protocol are reported elsewhere [21], but are summarised below.

### Study sample and data collection

For the baseline sample at T1, participants were selected from the 2016/2017 adult NZHS participants who had consented to be re-contacted for future research (within a two year period) and with sufficient data on study variables (exposure, confounders and baseline health) and contact details (n = 11,775). Individuals in the exposed group were sampled based on their reported experience of racism (ever), stratified by broad ethnic groupings (Māori, Pacific, Asian, European and Other). All exposed NZHS participants were invited to participate in the follow-up survey (n = 2099), with equal numbers of unexposed participants selected using propensity score matching (propensity to experience racism, based on key available predictive factors: full details in [21]).

Individuals selected for follow-up were sent an invitation letter and initially given the choice to participate by paper or web-based questionnaire. Individuals not responding following reminders (postcard plus text or email reminder) were contacted by telephone to ask if they would participate using computer-assisted telephone interview (CATI). Those who agreed to participate completed a short questionnaire covering current health status (mental and physical health) and recent health-service utilisation (unmet need and experiences with healthcare). The fieldwork was conducted by Research New Zealand. Participants were offered a NZ $20 voucher to compensate for their time.

A total of 3601 invitations were sent in four stages (initial small-scale launch, followed by three tranches of invitations) between 12 July 2018 and 8 October 2018. This allowed the management of fieldwork capacity and the tracking of follow-up response rates by exposure groups and ethnic grouping. Due to high recruitment in the first two tranches, invitations were not sent to the remaining European participants (n = 596). A total of 2010 participants responded (follow-up response rate = 55.8%). Respondents were evenly distributed across exposed (experience of racism, n = 1012) and non-exposed groups (no experience of racism, n = 998), reflecting the matched sampling of participants based on experience of racism, with a similar response rate between groups. Participants included 723 Māori (follow-up response rate = 50%), 99 Pacific (follow-up response rate = 41%), 332 Asian (follow-up response rate = 54%), 837 European (follow-up response rate = 67%) and 19 people from ‘Other’ ethnic groups (follow-up response rate = 50%). Key characteristics used in propensity score matching were also evenly distributed across exposed and non-exposed groups (Additional file 1).

### Study measures

The study protocol documents the sources of the outcome variable questions with a copy of the follow-up questionnaire (T2) [21]. The full 2016/2017 NZHS questionnaire that provided baseline data (T1) is also available [23].

### Exposure variables

Experience of racial discrimination was the exposure of interest, measured in the 2016/2017 NZHS using a series of five items asking participants about experience of an ethnically motivated (1) physical and/or (2) verbal attack, or unfair treatment due to their ethnicity in (3) health, (4) housing, or (5) work domains within the last 12 months or ever. Details of the questions and response options are in the study protocol [21]. For this analysis, exposure was defined as reporting experience of racism in any of the five domains in any time period (within last 12 months, or longer than 12 months ago).

### Outcome variables

A series of questions on health service use and experience were included in the follow-up questionnaire (T2), covering unmet healthcare need, satisfaction with their usual medical centre, and experiences with general practitioners (GPs - primary care doctor), as summarised in Table 1. *Unmet need for healthcare* was asked about for the last 12 months and included general unmet healthcare need, unmet need for mental healthcare, and unmet need for a GP.

**Table 1:** Health service outcome variables, response options and categories in logistic regression

Health care measure	Question	Response options	Logistic regression (categorical and/or ordinal)
**Unmet need**			
General unmet need	In the last 12 months, was there ever a time that you needed health care but could not get it?	Yes No Did not need healthcare Don’t know (DK)/refused	Categorical (yes vs. no) DK/refused classified as missing
Unmet need – mental health	In the past 12 months, did you ever feel that you needed professional help for your emotions, stress, mental health, or substance use, but you didn’t receive that help? This could have been because of personal reasons (for example it cost too much) or reasons you couldn’t control (for example no appointments available).	Yes No DK/Refused	Categorical (yes vs. no) DK/refused classified as missing
Unmet need – primary care	In the last 12 months, has there been any time when you needed to see a GP about your own health, but didn’t get to see any doctor at all? With a follow-up question of: How many times has this happened in the past 12 months?	Yes No DK/R Ordinal responses: 0, 1, 2, 3–5, > 5, DK/R	Categorical (yes vs. no) DK/refused classified as missing Ordinal models use frequency of how many times this has happened
**Satisfaction (Asked only of participants with a usual medical centre)**			
Satisfaction with medical centre	Overall, how satisfied are you with the care you got at your usual medical centre in the last 12 months? This includes all staff not just the GP.	Very satisfied Satisfied Neither satisfied or dissatisfied Dissatisfied Very dissatisfied NA – I have not been to my usual medical centre in the last 12 months	Categorical analysis (very dissatisfied/dissatisfied/neither satisfied or dissatisfied vs. very satisfied/satisfied) NA classified as missing (not in scope for question) Analysed as ordinal variable
**Patient experiences (only asked if had been to GP in the last 12 months)**			
Explaining health conditions	Thinking about your last visit to a GP… How good was the doctor at explaining your health conditions and treatments in a way that you could understand?	Very good Good Neither good or bad Poor Very Poor Doesn’t apply	Categorical analysis (very poor/poor/neither good or bad vs. very good/good) Also analysed as ordinal variable Doesn’t apply classified as missing
Involvement in decisions about care	Thinking about your last visit to a GP… How good was the doctor at involving you in decisions about your care, such as discussing different treatment options?	Very good Good Neither good or bad Poor Very Poor Doesn’t apply	Categorical analysis (very poor/poor/neither good or bad vs. very good/good) Also analysed as ordinal variable Doesn’t apply classified as missing
Being treated with respect and dignity	Thinking about your last visit to a GP… How good was the doctor at treating you with respect and dignity?	Very good Good Neither good or bad Poor Very Poor Doesn’t apply	Categorical analysis (very poor/poor/neither good or bad vs. very good/good) Also analysed as ordinal variable Doesn’t apply classified as missing
Confidence and trust in GP	Still thinking about your last visit to a GP…. Did you have confidence and trust in the last GP you saw?	Yes, definitely Yes, to some extent No, not at all	Categorical analysis (no, not at all/yes, to some extent vs. yes, definitely) Also analysed as ordinal variable

*For satisfaction and patient experience variables*, participants were instructed to answer these questions only if they had a usual medical center or had been to the GP in the last 12 months (as noted below) for the paper copy of the questionnaire. For online and telephone respondents, these questions were only asked for participants meeting these criteria. For consistency, analysis of respondents to the paper survey followed the questionnaire logic for the online and telephone surveys (i.e. individuals were not included in numerator/denominator for experience questions if they indicated they had not visited the GP in the last 12 months, even if they had answered the subsequent questions about experience with their GP).

Participants were asked how satisfied they were with their usual medical centre in the last 12 months (covering all staff, not just the GP). Participants were also asked about their experiences of their last visit to a GP. This included how good the doctor was at: explaining health conditions and treatments; involving the participant in decisions about care; and treating the participant with respect and dignity. Additionally, participants were asked about their confidence and trust in the GP.

Outcome variables were conceptualised as discrete recent (last 12 months) events and therefore did not need to be available at baseline (T1).

### Other variables

Other potential confounding variables (previously shown to be associated with racism [13]) were sourced from participants’ baseline data in the NZHS at T1. The initial set of confounders were all included in the propensity score calculations used to sample NZHS respondents [21], and included age group (15–24, 25–34, 35–44, 45–54, 55–64, 65–74, 75 + years); gender (male, female - as only gender response options available); prioritised ethnic grouping (prioritised in the order: Māori, Pacific, Asian, Other, European) [24]; nativity (born in NZ, born overseas); education qualification (secondary qualification or above, no secondary qualification or above); and the small-area measure of deprivation NZDep13 as quintiles [25]. The propensity score modelling (and additional adjustment) also included interaction terms for nativity by ethnic grouping (to account for differential experiences for those born in NZ and those born overseas).

### Data analysis

Basic frequencies and percentages are provided for healthcare outcome variables for the total sample and by racism exposure status (Table 2). These percentages describe the patterning of outcomes in the study cohort, and should not be interpreted as representative estimates for the general population as due to the propensity score matching used in sampling, proportions are only representative of a specific population (i.e. the types of people exposed to racism). Therefore, the focus of the results is on presenting adjusted ORs as the best estimates of difference between the exposed and non-exposed groups.

**Table 2:** Frequency and proportion of participants reporting unmet need for healthcare, satisfaction with healthcare, and patient experiences in the last 12 months (at T2), overall and by experience of racism ‘ever’ (at T1)

Outcome and response level	Total (n = 2010)			Exposed to racism Ever (n = 1012)			Not exposed to racism (n = 998)	
	n	%		n	%		n	%
**Unmet need**								
Yes	335	16.8		205	20.4		130	13.1
No	1413	70.8		685	68.2		728	73.5
NA - did not need (coded as No)	248	12.4		115	11.4		133	13.4
Missing	14			7			7	
**Unmet need: Mental Health**								
Yes	314	16.2		184	18.8		130	13.6
No	1624	83.8		795	81.2		829	86.4
Missing	72			33			39	
**Unmet need: General Practitioner (GP)**								
Yes	344	17.9		203	20.8		141	14.8
No	1582	82.1		771	79.2		811	85.2
Missing	84			38			46	
**Frequency of unmet need (GP)**								
0	1582	82.3		771	79.3		811	85.3
1	97	5.0		53	5.5		44	4.6
2	123	6.4		74	7.6		49	5.2
3–5	89	4.6		54	5.6		35	3.7
> 5	32	1.7		20	2.1		12	1.3
Missing	87			40			47	
**Satisfied with usual care**								
V satisfied	704	40.1		327	36.5		377	43.9
Satisfied	731	41.7		384	42.9		347	40.4
Neither	189	10.8		117	13.1		72	8.4
Dissatisfied	93	5.3		51	5.7		42	4.9
V dissatisfied	38	2.2		17	1.9		21	2.4
(NA -- has not visited usual medical centre)	73			33			40	
(NA -- no usual medical centre)	123			55			68	
Missing	59			28			31	
**Doctor explaining health conditions and treatments**								
V good	835	55.9		409	51.5		426	60.9
Good	460	30.8		267	33.6		193	27.6
Neither good or bad	132	8.8		81	10.2		51	7.3
Poor	42	2.8		21	2.6		21	3.0
V poor	25	1.7		16	2.0		9	1.3
(NA -- no visit in last 12 m)	403			169			234	
(NA – didn’t apply to last visit)	27			9			18	
Missing	86			40			46	
**Involving in decisions about care**								
V good	737	50.3		357	45.7		380	55.6
Good	453	30.9		255	32.6		198	29.0
Neither good or bad	181	12.4		114	14.6		67	9.8
Poor	74	5.1		42	5.4		32	4.7
V poor	20	1.4		14	1.8		6	0.9
(NA -- no visit in last 12 m)	403			169			234	
(NA – didn’t apply to last visit)	57			22			35	
Missing	85			39			46	
**Treated with respect and dignity**								
V good	1036	68.3		522	65.0		514	72.1
Good	364	24.0		217	27.0		147	20.6
Neither good or bad	84	5.5		47	5.9		37	5.2
Poor	27	1.8		16	2.0		11	1.5
V poor	5	0.3		1	0.1		4	0.6
(NA -- no visit in last 12 m)	403			169			234	
(NA – didn’t apply to last visit)	8			2			6	
Missing	83			38			45	
**Confidence and trust in GP**								
Yes, definitely	1088	71.4		540	67.2		548	76.2
Yes, to some extent	380	25.0		232	28.9		148	20.6
No	55	3.6		32	4.0		23	3.2
(NA -- no visit in last 12 m)	403			169			234	
Missing	84			39			45	

The degree of missing data is indicated for each outcome variable. For patient experience variables, those who were out of scope/ineligible for that question were excluded from the analysis as these were only answered by those who had a usual medical centre (for satisfaction) and those who had a visit to a GP in the last 12 months (for other questions). For these questions, percentages are reported for the denominator of those who were eligible to answer the question.

Logistic regression models examining the association between experience of racism (at T1) and health service use and experience (at T2) used doubly-robust estimation [26] to account for the propensity scores used in the sampling step (using inverse probability of treatment weights, IPTW) with additional adjustment for confounders by including them in the model to allow for any residual imbalance in confounders in the achieved sample. This adjustment included all covariates included in the propensity score model as noted above (age, gender, ethnicity, nativity, education qualification, and NZDep13).

As the relationship between racism and negative healthcare measures may be confounded by health status, analysis of the association of racism with unmet need was additionally adjusted for baseline self-rated health (excellent, very good, good, fair, poor) or mental health when analysing unmet need for mental healthcare (adjusting for K10 [27] as a continuous score). Analysis of satisfaction and experience of healthcare (satisfaction with medical centre, explanation of health conditions, involvement in decisions, treated with respect and dignity, confidence and trust in GP) were not adjusted for health status as we did not think there was a strong conceptual basis for considering health status as a confounder of the association from general racism exposure to healthcare experience.

Analysis was conducted using R 4.0 (R Institute, Vienna, Austria). Logistic regression models were conducted using the survey package [28] to incorporate propensity score weights and adjustment for covariates, as noted above. Weights were constructed as inverse probability of treatment weights (IPTW, where ‘treated’ in the usual usage corresponds to ‘exposed’ in this observational study). These propensity score weights were used in the model refined using an Average Treatment in the Treated (ATT) estimand (again where ‘treated’ in the usual usage is here exposure to racism), in order to estimate the *average impact of exposure to racism* amongst the kinds of people typically exposed to racism [29].

As the data available for analysis were achieved following the propensity score sampling phase, and hence are effectively pre-matched on key confounders (hence unadjusted estimates cannot be determined from this study), only the doubly-robust, fully-adjusted odds ratios (aOR) from the IPTW analysis are presented in results.

For some outcome variables (general unmet healthcare need, unmet need for mental healthcare) the primary regression models were undertaken using only binary logistic regression using the cutpoints described in Table 1; analyses for other outcome variables are reported treating the outcomes as binary outcomes (as above) and also as ordinal outcomes by applying ordinal logistic regression models. Odds ratios from ordinal models can be considered as conceptually equivalent to the relative odds of exposed group respondents reporting a higher-level of the response than the unexposed group (across all potential response option levels).

## Results

Table 2 shows the number and proportion of respondents reporting unmet healthcare need in the last 12 months, for the whole sample and also amongst those who report experiencing racism ‘ever’, and those reporting no experience of racism. Overall, reporting of unmet need was relatively low across all unmet need variables (e.g. 16.8% for any unmet need). Respondents who had experienced racism at T1 had higher reporting at T2 of unmet need in the last 12 months than those who reported no experience of racism at T1 (aOR = 1.71, 95% CI 1.31, 2.23) (Table 3). Similar patterns were seen for unmet need for mental healthcare (aOR = 1.36, 95% CI 1.02, 1.79) and unmet need for a GP (aOR = 1.53, 95% CI 1.18, 1.99). Analysis of frequency of unmet need for a GP (number of occasions, treated as zero for those reporting no unmet need) displayed a similar pattern (ordinal model aOR = 1.54, 95% CI 1.18, 2.00).

**Table 3:** Logistic regression estimates for the association between experience of racism ‘ever’ (at T1) and increased likelihood (at T2) of unmet need for healthcare in the last 12 months and negative experience

	Categorical models		Ordinal models	
HSU variable	OR	95% CI	OR	95% CI
**Unmet need (yes)**				
Unmet need for any healthcare (n = 1996)	1.71	(1.31, 2.23)		
Unmet need for mental health care* (n = 1938)	1.36	(1.02, 1.79)		
Unmet need for GP Categorical (yes/no) (n = 1926) Ordinal (frequency of unmet need) (n = 1923)	1.53	(1.18, 1.99)	1.54	(1.18, 2.00)
**Experience of healthcare** **(negative)**				
Satisfaction with usual medical centre (n = 1755)	1.41	(1.10, 1.81)	1.37	(1.14, 1.63)
Doctor explaining health conditions and treatments (n = 1494)	1.35	(0.99, 1.84)	1.44	(1.17, 1.76)
Doctor involving patient in decision making about their care (n = 1465)	1.60	(1.22, 2.09)	1.51	(1.24, 1.84)
Treated with respect and dignity (n = 1516)	1.14	(0.77, 1.70)	1.36	(1.09, 1.70)
Confidence and trust in GP (n = 1523)	1.62	(1.29, 2.04)	1.60	(1.27, 2.01)

Note: all models adjusted for age, gender, ethnicity, nativity, education, employment, NZDep2013, nativity x ethnicity. Unmet need models are also adjusted for underlying health (self-rated health, except unmet need for mental health care* which is adjusted for K10)

Of those people with a usual medical centre and visit in the last 12 months (1755/1951 = 90% of sample excluding missing), most reported being satisfied with their usual medical centre in the last 12 months (81.8% very satisfied/satisfied; and 18.3% reported being very dissatisfied, dissatisfied, or neither satisfied nor dissatisfied) (Table 2). However, people who reported racism at T1 reported a higher combined proportion of neutral or dissatisfaction responses (aOR = 1.41, 95% CI 1.10, 1.81) (Table 3). The ordinal analysis also suggested the response profile for those reporting racism at T1 was shifted more towards the dissatisfied end of the scale (ordinal aOR = 1.37, 95% CI 1.14, 1.63).

Other patient experience variables were analysed among a smaller number of respondents, as these questions required that participants had a visit to a GP in the last 12 months to answer these questions. Most people had very positive experiences at their last visit to a GP within the preceding 12 months (see Table 2 for total sample counts and proportions). However, among those who had visited a GP in the last 12 months, positive experiences tended to be more common among those who had not experienced racism at T1 than those who had, with the exception of reporting being treated with respect and dignity, which was similar by exposure status. For these experiences, aORs (Table 3) from binary logistic regression generally indicated higher levels of reporting neutral or negative experiences (neutral, poor, or very poor) amongst those reporting experience of racism at T1 (e.g. doctor’s explanation of conditions/treatments: aOR = 1.35, 95% CI 0.99, 1.84; treated with respect and dignity, aOR = 1.14, 95% CI 0.77, 1.70). Analysis using ordinal models that did not impose binary cutpoints provided more clear-cut evidence that responses were shifted more towards the negative side of the scale for those reporting experience of racism at T1 (doctor’s explanation: ordinal aOR = 1.44, 95% CI 1.17, 1.76; treated with respect and dignity, ordinal aOR = 1.36, 95% CI 1.09, 1.70).

## Discussion

This study demonstrates using a high-quality prospective design that ever experiencing racism is linked to subsequent higher unmet need, lower satisfaction with healthcare and generally poorer experiences with visits to a GP. It is important to understand these findings in the context of ethnic inequities in exposure to racism, whereby people from non-European ethnic groups (e.g. Māori, Pacific, Asian) report higher experience of racial discrimination than people from the broad European grouping (Additional file 2; [13]) alongside the potential contribution of this to inequities in healthcare use and experience.

These findings are consistent with the broader international literature that shows links between racism and negative healthcare measures, particularly measures of unmet need for healthcare and poorer satisfaction and patient experiences [9]. This study addresses a gap in current literature, with most studies being cross-sectional [9]. An important strength of this study is the longitudinal prospective design, particularly the measurement of exposure to racism (T1:2016/17 NZHS) prior to healthcare measures (T2: 1–2 year follow-up), adding to the strength of evidence that racism is a potentially causal factor in determining poorer access to healthcare and poorer quality of care.

While this study demonstrates a prospective association between experience of racism and adverse healthcare measures of unmet need, lower satisfaction and worse experiences, it does not show the specific mechanisms by which racism impacts healthcare. As a complex system, the mechanisms by which racism may impact healthcare are likely to be multiple, with both direct and indirect pathways that can operate at both institutional and individual provider levels [9, 21, 30], with the potential for racism to “… impact the quality of healthcare, how individuals access and use health services, and experiences and perceptions of healthcare” [9]. For example, experiences of racism (both within healthcare and more broadly) may lead to higher unmet need via mechanisms such as socioeconomic status, increased health need and reduced access to healthcare providers [9, 21, 31]. In this study, we adjusted for health status (e.g. self-rated health or mental health) in the examination of racism and unmet need for healthcare, and still show an independent association, suggesting mechanisms other than increased health need. We also demonstrated an association between experience of racism and lower confidence and trust in GPs, which has the potential to affect access to and experience of healthcare. Benjamin [32] reminds us that measures of trust among individuals reflect the “trustworthiness” (or not) of providers/systems and in NZ is supported by extensive evidence of ethnic healthcare inequities [33].

The mechanisms linking experience of racism and lower satisfaction and negative experiences are similarly likely to be complex and multidimensional, encompassing poorer quality care as well as patient perceptions of care [9, 30]. For example, discriminatory treatment may lead to lower satisfaction and poorer patient experiences; racism experienced outside the health system may lead to lower trust in healthcare providers; experience of racism may influence patient perceptions of care as negative; and, experience of racism more broadly can elicit stereotype threat that can negatively influence healthcare encounters [9, 30].

The complex and multifaceted potential mechanisms by which experience of racism can impact healthcare cannot be determined from quantitative studies like this one that only include measures of exposure and outcome. These individual experiences also exist within the broader system of racism that operates at institutional and societal levels, as well as at the level of individuals. We are conscious of not wanting to over-interpret findings or focus too heavily on individuals, particularly those exposed to racism, as this may inadvertently shift the framing of the problem to the level of individuals exposed to racism rather than the broader system of racism, which has previously been described as deflecting “discourse from the ways in which group stereotypes and institutional arrangements are products of racism and serve to reinforce racial inequality” [31, p202]. Instead, interventions that address the distribution of power implicit in racism require that anti-racism actions seek to eliminate racism and that health systems and providers understand their roles and responsibilities in relation to this. Such interventions also need to operate at multiple levels [34], and across different sectors to realise meaningful and long-lasting change.

In addition to the strengths of a prospective study, this study also adds to the limited international studies undertaken at a national population level, with sufficient data to understand the impacts of racism for Indigenous populations. Methodologically, the propensity matching approach provided an efficient and effective means to minimise potential confounding. However, this approach is limited to adjusting for pre-planned variables that existed in the baseline NZHS data, and there is still potential for residual confounding if confounders were inadequately measured or not measured at all. While there may be other factors impacting on access to care that do not relate to experience of racism, our design for comparison of those exposed/unexposed to racism likely accounts for major confounders that might be expected to drive differential access to healthcare. Secondly, the multimodal survey design allowed us to maximise response rates, although opening the potential for differential responding by modality. This was mitigated prior to follow-up data collection through careful questionnaire design to ensure alignment of questions and consistent handling of responses across modalities e.g. in-built skips in online and telephone modalities were applied to paper questionnaire responses. The distribution of modality was also similar across exposure groups. Considering outcome variables ordinally also allowed for examination of more subtle impacts of racism on variables where this was appropriate. This analysis of HSU outcomes with ordinal logistic regression returned similar patterns to the binary outcome analysis, but avoided the potential to be determined by (sometimes) arbitrary decisions about where to place the cut-point in making a binary categorisation (e.g. the boundary between satisfied and dissatisfied response categories). This has additional utility as the distribution of these outcome variables may reflect a general downgrading of responses in the exposed group (shifting more towards lower satisfaction) without necessarily crossing these arbitrary boundaries.

There are several limitations that should be considered in interpreting these findings. Firstly, the timeframe to follow-up was relatively short, between one to two years. It is possible that the impacts of racism on healthcare measures may be different at different lead-times. This is seen in health status outcomes, where the lead-time of racism on mental health outcomes is shorter than physical health outcomes [9, 35]. In addition, the follow-up time is for a composite one to two year follow-up period, rather than a fixed interval, yielding variable follow-up time among participants (although this is not different between exposed and unexposed). We were unable to undertake analyses stratified by ethnicity as participation across ethnic groups was largely driven by who had participated in the NZHS from which we sampled, limiting our ability to report results further stratified by ethnicity. We note however that a previous cross-sectional study suggests that different strengths of associations were not a major feature for individual ethnic categories [13]. Furthermore, the ethnic categories that we stratified for in sampling do not represent individual ethnicities (apart from Māori) but rather are broad groupings of diverse populations e.g. the Pacific, Asian and European categories are made up of multiple ethnic groups. Additionally, it is unclear how these relationships may be affected by the COVID-19 pandemic and the increase in telehealth consultations that occurred after the data collection period of this study. Further research could examine whether these associations are found in analyses of recent experiences of racism (rather than ever) and for racism experienced in healthcare settings specifically. This was not possible in this study due to relatively small numbers for these potential analyses, and the propensity score sampling step matching groups based on ever experience of racism. It was also not possible to examine impact of experience of racism by a health professional separately to other forms of racism due to small numbers of participants reporting this exposure. Cross-sectional analysis of the NZHS has shown racism by a health professional to have stronger associations with negative healthcare measures [31], and so we might have expected a similar phenomenon in prospective results as well.

## Conclusion

This study adds to the growing evidence that experience of racism both within healthcare and in society more broadly potentially leads to poorer healthcare through higher unmet need, lower satisfaction and more negative experiences of healthcare. It is important that healthcare providers and organisations prevent racism within their working environments and understand and mitigate the negative impacts of racism on patient healthcare, at both individual provider and organisational levels. This is an important contribution that the health system can undertake to complement and support broader efforts to eliminate racism societally.

### Electronic supplementary material

Below is the link to the electronic supplementary material.

Additional file 1: Distribution of key characteristics between exposed (ever experience of racism) and not exposed (no experience of racism) groups at follow-up (T2)

Additional file 2: Prevalence of self-reported experience of racial discrimination ?ever? by ethnic group, 2016/17 NZHS

## Data Availability

The NZHS 2016/17 data used as the baseline for the study described in this protocol is available to approved researchers subject to the New Zealand Ministry of Health’s Survey Microdata Access agreement https://www.health.govt.nz/nz-health-statistics/national-collections-and-surveys/surveys/access-survey-microdata. Data from the follow-up study are not able to be shared as this is a requirement of the ethics approval for this study. For queries about data availability please contact Ricci Harris (ricci.harris@otago.ac.nz).

## Abbreviations

95% CI: 95% confidence interval
aOR: Adjusted Odds Ratio
ATT: Average Treatment in the Treated
CATI: Computer-assisted telephone interview
GP: General Practitioner
IPTW: Inverse probability of treatment weights
K10: Kessler-10
NZ: Aotearoa New Zealand
NZDep13: New Zealand Deprivation Index 2013
NZHS New: Zealand Health Survey
SES: Socioeconomic status
T1: Time point 1
T2: Time point 2
