# Supplementary material for: The impact of racism on subsequent healthcare use and experiences for adult New Zealanders: a prospective cohort study
Source: BMC Public Health. 2024 Jan 9;24:136. doi: 10.1186/s12889-023-17603-6 (PMC10777617; doi:10.1186/s12889-023-17603-6)
Supplement: Supplementary file 1 — Additional file 1: Distribution of key characteristics between exposed (ever experience of racism) and not exposed (no experience of racism) groups at follow-up (T2) [file 12889_2023_17603_MOESM1_ESM.docx]

**Additional file 1: Distribution of key characteristics between exposed (ever experience of racism) and not exposed (no experience of racism) groups at follow-up (T2)**

| **Key characteristic** | **Not exposed to racism**  **N=998** | **Exposed to racism (ever)**  **N=1012** | **Total**  **N=2010** |
| --- | --- | --- | --- |
| **Ethnicity** | **n (%)** |  |  |
| European | 419 (42.0) | 418 (41.3) | 837 (41.6) |
| Māori | 373 (37.4) | 350 (34.6) | 723 (36.0) |
| Pacific | 41 (4.1) | 58 (5.7) | 99 (4.9) |
| Asian | 156 (15.6) | 176 (17.4) | 332 (16.5) |
| MELAA/Other | 9 (0.9) | 10 (1.0) | 19 (0.9) |
|  |  |  |  |
| **Nativity** |  |  |  |
| Born in NZ | 721 (72.2) | 713 (70.5) | 1434 (71.3) |
| Born Overseas | 277 (27.8) | 299 (29.5) | 576 (28.7) |
|  |  |  |  |
| **Gender** |  |  |  |
| Men | 409 (41) | 468 (46.2) | 877 (43.6) |
| Women | 589 (59) | 544 (53.8) | 1133 (56.4) |
|  |  |  |  |
| **Age-group (yrs)** |  |  |  |
| 15-24 | 75 (7.5) | 54 (5.3) | 129 (6.4) |
| 25-34 | 154 (15.4) | 158 (15.6) | 312 (15.5) |
| 35-44 | 187 (18.7) | 203 (20.1) | 390 (19.4) |
| 45-54 | 193 (19.3) | 193 (19.1) | 386 (19.2) |
| 55-64 | 188 (18.8) | 190 (18.8) | 378 (18.8) |
| 65-74 | 152 (15.2) | 155 (15.3) | 307 (15.3) |
| 75+ | 49 (4.9) | 59 (5.8) | 108 (5.4) |
|  |  |  |  |
| **Education qualification** |  |  |  |
| Less than upper secondary | 215 (21.5) | 202 (20.0) | 417 (20.7) |
| Upper secondary | 271 (27.2) | 265 (26.2) | 536 (26.7) |
| Tertiary | 461 (46.2) | 471 (46.5) | 932 (46.4) |
| Other | 51 (5.1) | 74 (7.3) | 125 (6.2) |
|  |  |  |  |
| **Employment** |  |  |  |
| Working | 602 (60.3) | 600 (59.3) | 1202 (59.8) |
| Looking for work | 49 (4.9) | 51 (5.0) | 100 (5.0) |
| Not in Labour Force | 189 (18.9) | 177 (17.5) | 366 (18.2) |
| Not in Labour Force (age 65+ yrs) | 151 (15.1) | 171 (16.9) | 322 (16.0) |
|  |  |  |  |
| **NZDep13 quintile** |  |  |  |
| 1 (least deprived) | 102 (10.2) | 141 (13.9) | 243 (12.1) |
| 2 | 148 (14.8) | 143 (14.1) | 291 (14.5) |
| 3 | 208 (20.8) | 182 (18.0) | 390 (19.4) |
| 4 | 227 (22.7) | 227 (22.4) | 454 (22.6) |
| 5 (most deprived) | 313 (31.4) | 319 (31.5) | 632 (31.4) |
|  |  |  |  |
| **Self-rated health*** |  |  |  |
| Excellent | 135 (13.5) | 106 (10.5) | 241 (12.0) |
| Very good | 391 (39.2) | 340 (33.6) | 731 (36.4) |
| Good | 330 (33.1) | 377 (37.3) | 707 (35.2) |
| Fair | 106 (10.6) | 136 (13.4) | 242 (12.0) |
| Poor | 36 (3.6) | 53 (5.2) | 89 (4.4) |
|  |  |  |  |
| **K10 (psychological distress)*** | **Mean (SD)** | **Mean (SD)** | **Mean (SD)** |
|  | 3.51 (5.20) | 4.75 (6.85) | 4.03 (5.61) |
|  |  |  |  |

Note: *not included in propensity score matching, but included in some regression analyses
