# Supplementary material for: The impact of racism on subsequent healthcare use and experiences for adult New Zealanders: a prospective cohort study
Source: BMC Public Health. 2024 Jan 9;24:136. doi: 10.1186/s12889-023-17603-6 (PMC10777617; doi:10.1186/s12889-023-17603-6)
Supplement: Supplementary file 2 — Additional file 2: Prevalence of self-reported experience of racial discrimination ?ever? by ethnic group, 2016/17 NZHS [file 12889_2023_17603_MOESM2_ESM.docx]

**Additional file 2: Prevalence of self-reported experience of racial discrimination ‘ever’ by ethnic group, 2016/17 NZHS**

To provide context of the patterning of experience of racism, prevalence estiamtes were calculated by ethnicity for the 2016/17 NZHS (below table). The estimates in this table are the population prevalences for experience of racism by ethnic category from all particpants in the baseline 2016/17 New Zealand Health Survey. Prevalences are weighted proportions that account for the complex survey design in order to calculate representative estimates for the population. The methods used are similar to those published elsewhere (Harris et al. 2018). Experience of racism is not experienced evenly. Prevalence estimates show that non-European ethnic groups are more likely to report experiences of all forms of racism measured in the 2016/17 NZHS.

| **Racial discrimination measures** |  | **Māori** |  | **Pacific** |  | **Asian** |  | **‘Other’** |  | **European** |  |
| --- | --- | --- | --- | --- | --- | --- | --- | --- | --- | --- | --- |
|  |  | **N=2719** |  | **N=687** |  | **N=1251** |  | **N=119** |  | **N=8722** |  |
|  |  | % | 95% CI | % | 95% CI | % | 95% CI | % | 95% CI | % | 95% CI |
|  |  |  |  |  |  |  |  |  |  |  |  |
| Personal attack | Verbal | 22.8 | (20.8, 25.0) | 13.6 | (10.9, 16.8) | 24.7 | (21.7, 28.0) | 14.2 | (8.8, 22.2) | 10.6 | (9.7, 11.6) |
|  | Physical | 7.6 | (6.3, 9.1) | 3.2 | (2.0, 5.1) | 3.1 | (2.2, 4.4) | 4.1 | (1.5, 11.1) | 3.1 | (2.7, 3.6) |
| Unfair treatment |  |  |  |  |  |  |  |  |  |  |  |
|  | Health | 5.6 | (4.6, 6.8) | 5.0 | (3.1, 8.0) | 3.5 | (2.5, 5.0) | 5.1 | (2.0, 12.3) | 1.5 | (1.2, 1.9) |
|  | Work | 6.9 | (5.8, 8.1) | 5.9 | (4.1, 8.4) | 8.2 | (6.4, 10.4) | 7.6 | (3.7, 15.2) | 2.0 | (1.6, 2.5) |
|  | Housing | 7.2 | (6.1, 8.6) | 4.7 | (3.2, 6.9) | 4.9 | (3.6, 6.6) | 4.0 | (1.6, 9.7) | 0.5 | (0.4, 0.7) |
|  |  |  |  |  |  |  |  |  |  |  |  |
| Any experience |  | 29.5 | (27.3, 31.8) | 20.9 | (17.4, 24.9) | 31.0 | (27.9, 34.4) | 19.5 | (12.8, 28.6) | 13.4 | (12.3, 14.5) |
|  |  |  |  |  |  |  |  |  |  |  |  |
| Multiple experiences |  |  |  |  |  |  |  |  |  |  |  |
|  | Reports 1 | 16.8 | (15.1, 18.7) | 13.6 | (10.9, 16.9) | 20.7 | (18.0, 23.8) | 7.7 | (4.0, 14.4) | 9.6 | (8.8, 10.6) |
|  | Reports 2+ | 12.6 | (11.0, 14.5) | 7.3 | (5.1, 10.2) | 10.3 | (8.3, 12.7) | 11.8 | (6.5, 20.4) | 3.7 | (3.2, 4.3) |
|  |  |  |  |  |  |  |  |  |  |  |  |

Reference: Harris RB, Stanley J, Cormack DM. Racism and health in New Zealand: Prevalence over time and associations between recent experience of racism and health and wellbeing measures using national survey data. PloS one. 2018 May 3;13(5):e0196476.
